# Supplementary material for: Memory, switches, and an OR-port through bistability in chemically fueled crystals
Source: Nat Commun. 2022 May 20;13:2816. doi: 10.1038/s41467-022-30424-2 (PMC9122941; doi:10.1038/s41467-022-30424-2)
Supplement: Supplementary file 1 — Supplementary Information [file 41467_2022_30424_MOESM1_ESM.pdf]

Supplementary Information

Memory, switches, and an OR-port through bistability in chemically fueled crystals

Fabian Schnitter[1], Benedikt Rieß[1], Christian Jandl[2], Job Boekhoven[1,3,*]

1 Department of Chemistry, Technical University of Munich, Lichtenbergstrasse 4, 85748 Garching, Germany.

2 Catalysis Research Centre, Technical University of Munich, Lichtenbergstrasse 4, 85748 Garching, Germany

3 Institute for Advanced Study, Technical University of Munich, Lichtenbergstrasse 2a, 85748 Garching, Germany.

*Correspondence to: job.boekhoven@tum.de

Supplementary methods

Turbidity measurements.

The evolution of the turbidity during the reaction cycle is measured with an Arduino controlled setup (Supplementary Figure 5). The setup measures the scattering of 660 nm laser light under a 90° angle. The main advantage of such a setup compared to a commercial setup is the possibility to continuously stir the sample and connect it to inflow and outflow tubing. The scattering rate is measured at 10-second intervals. By using a smaller stirring plate, the setup can be put into an incubation chamber to control the temperature during the reaction cycle. The Arduino script is provided at: https://github.com/fabianschnitter/Script-turbidity-device.git.

Steady state experiments.

We used a continuously stirred tank reactor to create steady state concentrations. The reactor setup was made up of a 1.5 ml HPLC vial equipped with a micro stir bar put on a stir plate (600 rpm). The reactor was continuously supplied by a 0.3 $ml.min^{-1}$ inflow from a 150 mM precursor **1** or **3** stock solution (0.12 $ml.min^{-1}$ from a 37.5 mM precursor **2** stock solution). The inflow rate of EDC was equal to the inflow rate of precursor, but the stock concentration varied to apply different molar fluxes. The outflow rate was 0.6 $ml.min^{-1}$ (0.24 $ml.min^{-1}$ for precursor **2**). Aladdin AL-1000 syringe pumps from WPI were used equipped with 20 ml Braun Injekt syringes (ID = 20.10 mm). We used silicone tubing (OD = 1.8 mm, ID = 1.0 mm) and Braun Sterican needles (ID = 0.8 x 120 mm) for injection. For the outflow, a tygon tubing (OD = 3.2 mm, ID = 1.6 mm) was directly placed through a pre sliced septum of the HPLC vial in the reactor. A tubing diameter of 1.6 mm appeared to be best to homogeneously remove the solution with the crystals. At lower diameters, the tubing tended to clog, whereas larger diameters reduce the velocity which is necessary to remove the crystals. We tested the quality of constantly removing the crystal state anhydride by measuring the sum of concentration acid and anhydride inside the reaction vessel over time to exclude up concentration of the precursor (Supplementary Figure 4). For longer experiments larger 50 ml Braun Omnifix syringes (ID = 27.90 mm) were used. To measure the turbidity of the sample, the HPLC vial was placed in the home-made turbidity device. The temperature was controlled by placing the entire setup in an incubator. Measuring the reaction mixture's temperature was done by the In-outdoor Traceable thermometer purchased from VWR. The reaction vessel was supplied by the syringe pumps placed outside the incubator at controlled 21 °C ambient temperature. We prevented the reaction mixture to cool down by placing roughly 20 cm of tubing inside the incubator to allow the influx solution to approach the set incubator's temperature (Supplementary Figure 4). As a starting reaction mixture, 100 mM precursor **1**, 10 mM precursor **2,** or 150 mM precursor **3** was used. Thereby, addition of the 80 mM EDC spike (50 mM regarding **2**) before pumping

resulted in initial crystal or droplet formation. To measure the reaction cycle's concentrations, 30 µl aliquots were taken over time and quenched with a 200 mM benzylamine solution (1:1 dilution).

Determining the temperature dependence of S_{out} and S_{sat}.

The S_{out} and S_{sat} for several temperatures was determined in steady state experiments (Supplementary Fig. 18 and Fig. 1c). The S_{out} was determined by spiking the system beforehand applying a 10 mM.min^{-1} EDC and 30 mM.min^{-1} precursor **1** flux. This EDC flux did not sustain the crystalline on state which was shown by a decrease in scattering. We assumed a complete dissolution of the crystals at the timepoint reaching 0.15 scattering units. The anhydride concentration calculated by the kinetic model at this timepoint was taken as the S_{out}. The supersaturation concentration S_{sat} could not be measured directly as common methods like cooling down a solution of a known concentration is not possible. During the reaction cycle, anhydride is constantly produced and consumed and a change in temperature affects the steady state concentration. Furthermore, the induction time must be considered when directly measuring S_{sat}, which is the time between reaching a supersaturated solution and the onset of detectable nucleation[1]. Instead, we approached the supersaturation concentration by increasing the EDC flux and measuring the scattering of the system in steady state. For instance, at 21 °C, an EDC flux between 20 and 25 mM.min^{-1} evoked a crystal steady state detected by an increase in scattering. The corresponding anhydride steady state concentration was calculated by the kinetic model and taken as S_{sat} in the phase diagram.

Toggling the switch experiments.

The reactor was loaded with 1.5 ml water. The syringes were operated for 5 min to establish steady state concentrations (21 °C, 150 mM acid **1** stock, 100 mM EDC stock, inflow 0.3 ml.min^{-1}, respectively). For the activation, different amounts of EDC were added from a 2 M stock solution (30.0 µl, 22.5 µl, 15.0 µl, 7.50 µl), whereas the crystalline anhydride of **1** and the salts (NaCl: 438 mg, 263 mg, 88.0 mg; NaBr: 464 mg; NaI: 675 mg) were added as solids. As the crystalline anhydride was added in small amounts (1.6 mg, 1.1 mg, 0.54 mg, 0.11 mg), the reactor's volume was increased to 5 ml (150 mM acid stock, 100 mM EDC stock, inflow 1.0 ml.min^{-1}, respectively). Polystyrene (1.5 mg) was added as a control to ensure that the change in volume did not induce crystallization.

For the deactivation, 1.5 ml of 100 mM precursor **1** solution was initially activated with 40 mM EDC. After stabilizing the reactor for 5 min by pumping (21 °C, 150 mM acid **1** stock, 100 mM EDC stock, inflow 0.3 ml.min^{-1}, respectively), deactivation was done by adding various amounts of high concentrated stock solutions (5 M benzylamine: 90 µl, 60 µl, 45 µl; 33 wt% methylamine: 100 µl, 75 µl, 50 µl; 70 wt% ethylamine: 69 µl, 61 µl, 52 µl; 5 M 3-(dimethylamino)-

1-propylamine: 120 µl, 105 µl, 90 µl) to the reaction vial. The dilution was kept well below 10%. When switching the system several times, a fraction of the reaction solution was replaced by the deactivation stock to prevent volume expansion.

Pixel-display experiment.

The 3x3 pixel display reactor was 3D printed using the Form 2 from Formlabs applying a black photopolymer resin. After the print, the 3x3 pixel display was treated 20 min with isopropanol and cured for 5 min at 55 °C. Each of the nine reactors was filled 0.6 ml of 100 mM precursor **1**. Then, the flowrate in was set to 0.12 ml.min^{-1} (150 mM acid stock, 100 mM EDC stock) and the flowrate out to 0.24 ml.min^{-1}. To control 27 syringes simultaneously, two Aladdin AL-1600 and five Aladdin AL-1000 were used with 3D printed extensions to hold three syringes instead of one (Supplementary Fig. 24). The 3x3 pixel display was placed on a stir plate. Activation of the pixels was done by the addition of 12 µl of a 2 M EDC stock, whereas deactivation was done by replacing 36 µl of the reaction solution with 36 µl 5 M benzylamine solution. Timelapsed photographs in 10 seconds intervals were recorded by a Logitech BRIO webcam. Processing the photographs was done by imageJ extended by a fiji distribution. The 3D model 3x3 pixel display as well as the extension of the pumps can be found at: https://www.thingiverse.com/thing:4960191 and https://www.thingiverse.com/thing:4971601.

OR-port experiment.

A 1x3 reactor array was 3D printed as described above. Luer lock connectors were designed to easily connect the reactors with the inflow tubing. The outflow tubing is connected via a hole at the back of the reactors. At the bottom, cuvette micro stirrers from 2MAG are placed right beyond the reaction reactors. The flow from the input to the output reactor takes place via a 2 mm diameter hole. The reactor's turbidity is monitored by time lapsed photographs of the outflow tubings in 10 seconds intervals. Each reactor was filled with 1.5 ml water. Then, each reactor received an influx of 20 mM.min^{-1} EDC and 30 mM.min^{-1} precursor **1**. Specifically, the input reactors received 0.3 ml.min^{-1} of a 150 mM acid **1** stock and 0.3 ml.min^{-1} of a 100 mM EDC stock. From the input reactors, 0.4 ml.min^{-1} was withdrawn, and 0.2 ml.min^{-1} flowed into the output reactor. The output reactor received 0.1 ml.min^{-1} of a 450 mM precursor **1** stock and 0.1 ml.min^{-1} of a 300 mM EDC stock. A more detailed overview is given in Supplementary Figure 25. Moreover, the output reactor also received 0.2 ml.min^{-1} from each input reactor. Activation of the input reactors was done by the addition of 30 µL from a 2 M EDC stock or 263 mg NaCl. Deactivation was done by replacing 90 µl of the reaction solution with 90 µL 5 M benzylamine solution. The 3D model can be found at: https://www.thingiverse.com/thing:4971613.

Supplementary Figures

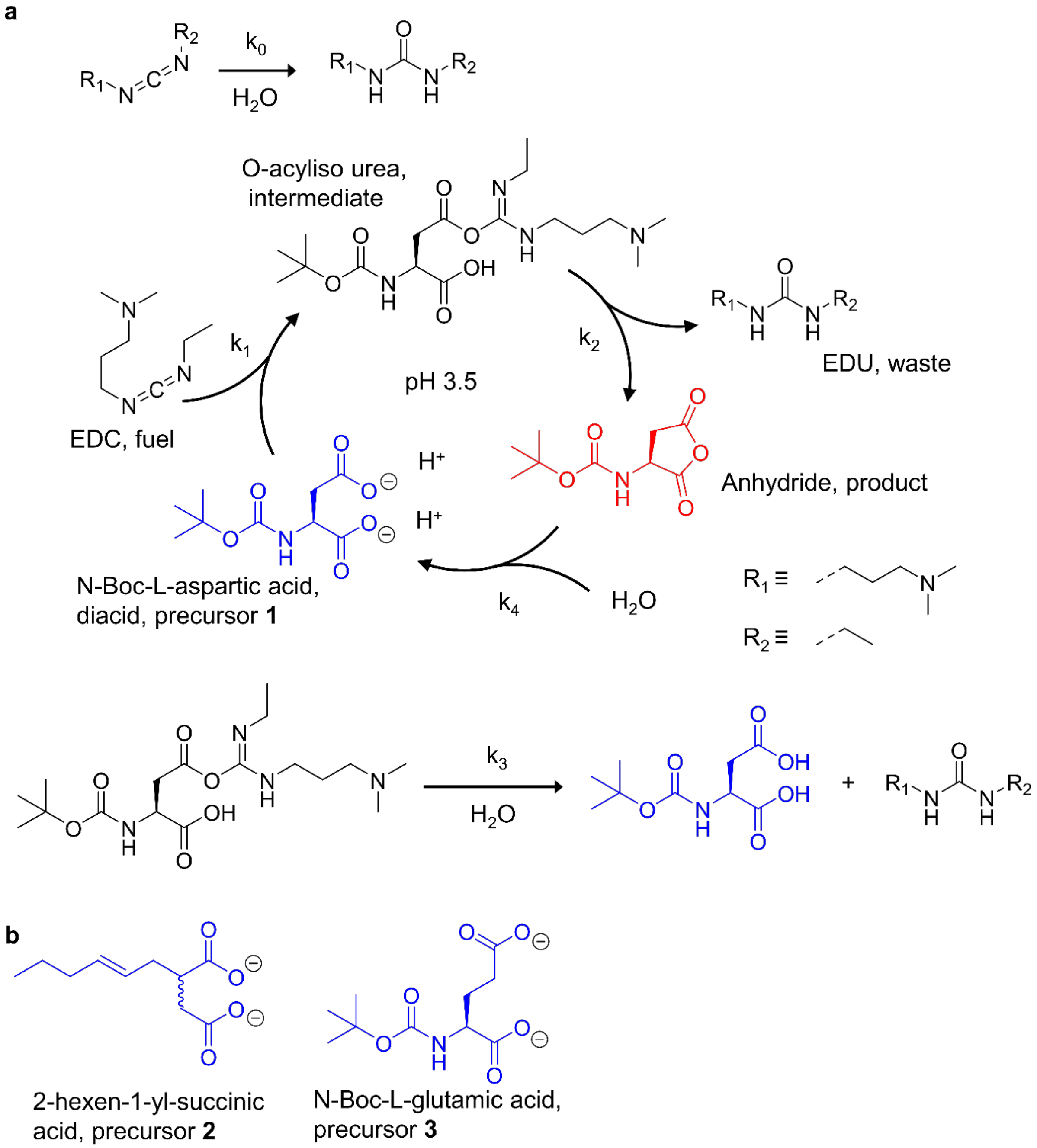

Supplementary Figure 1: The reactions involved in the chemical reaction cycle. a All reactions shown are simultaneously taking place at various rates. The direct hydration of the EDC fuel (k_0) was examined in previous work[2]. The start of the reaction cycle is the addition of one EDC molecule to the diacid precursor, forming a high energy intermediate (k_1). On releasing the waste product EDU, the intermediate further reacts to the active anhydride product (k_2). Furthermore, the intermediate decomposes in a side reaction at low rates to the precursor by direct hydrolysis (k_3). The deactivation reaction comprises the degeneration of the precursor driven by the anhydride hydrolysis (k_4). **b** Further diacids used as precursors.

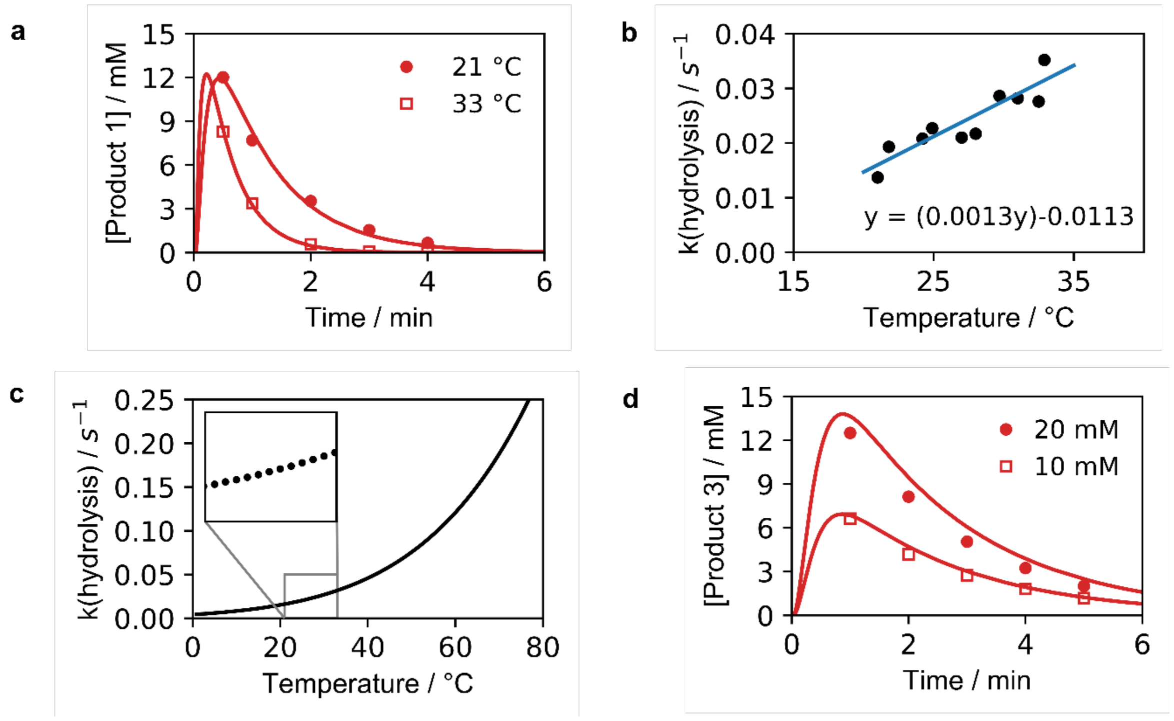

Supplementary Figure 2: Batch fueled experiments to extract deactivation rate constants. a The first order decay profile of anhydride product **1** hydrolysis over time at various temperatures. A 100 mM precursor solution was fueled with 20 mM EDC. The profiles at 21 °C and 33 °C are taken as exemplary curves for all temperatures measured. **b** The deactivation rate constants of **1** determined for different temperatures. **c** The effect of temperature on k_4 of **1** as calculated by the Arrhenius correlation. **d** The first order decay profile of 100 mM of precursor **3** when fueling with different amounts of EDC.

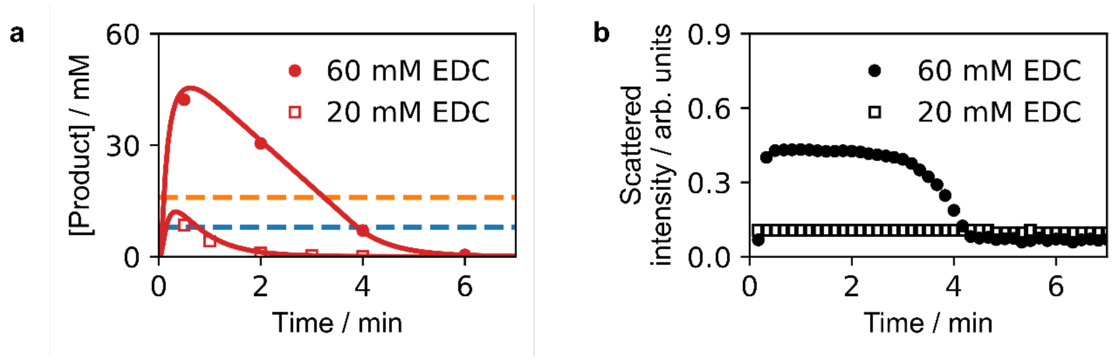

Supplementary Figure 3: The reaction cycle's response at 24 °C when adding different amounts of EDC fuel to 100 mM precursor 1[4]. a When adding 20 mM of EDC, the anhydride yield is below S_{sat} (orange line), whereas when increasing the EDC amount to 60 mM the anhydride concentration exceeds S_{sat}. **b** The measured scattering shows the presence of crystals when 60 mM fuel is added, whereas no increase in scattering was measured for 20 mM EDC addition.

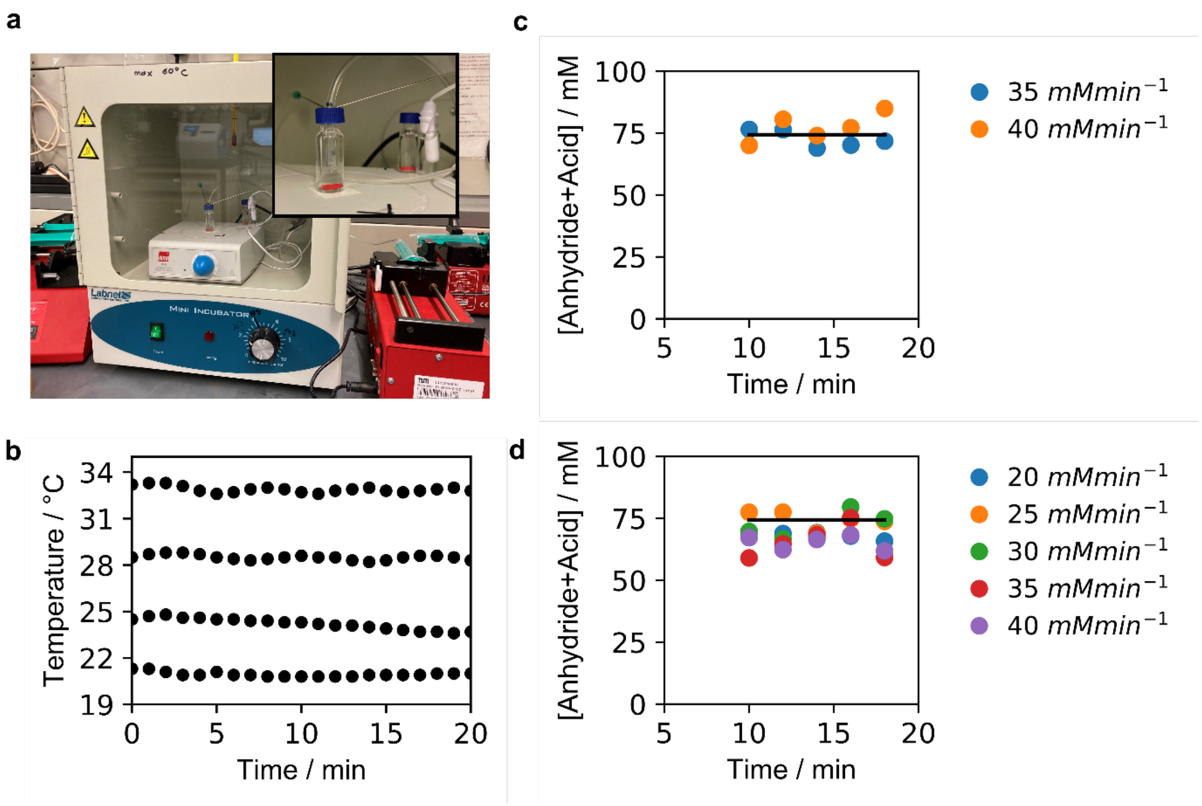

Supplementary Figure 4: Experimental setup of steady state experiments. a Photograph of the experimental setup. **b** Monitoring the temperature stability of the steady state experiments. **c**, **d** The sum of the concentration precursor **1** and the corresponding product over time. The concentration should be roughly constant and ensures that the crystals are not clogging the tubing or accumulate in the reactor. In the data in **c**, no spike was added. In contrast, an 80 mM EDC spike was added at **d**. The markers represent HPLC data, and the line represents the concentration predicted by the kinetic model.

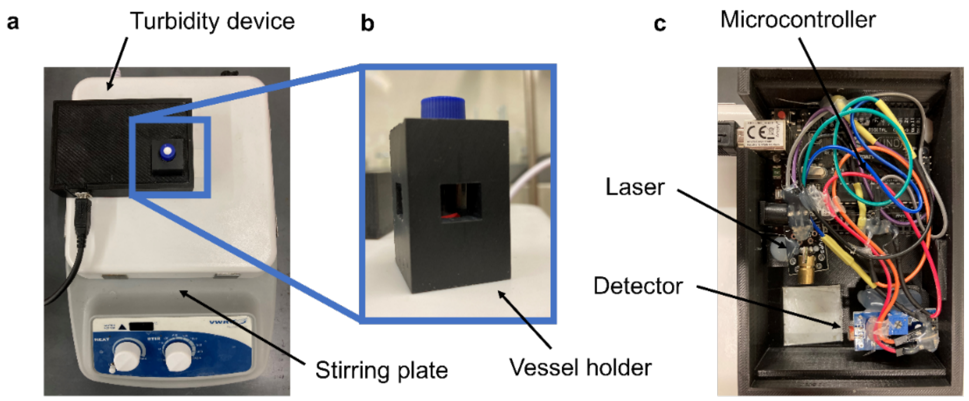

Supplementary Figure 5: The setup used to measure the reaction cycle's turbidity. a The device is placed on a stirring plate to homogeneously mix the crystals formed by the anhydride. **b** An HPLC vial was used as sample container, which is placed inside a 3D printed vessel holder. **c** The scattered light is measured under a 90° angle. The laser and detector are controlled by an Arduino microcontroller. The electronic components are covered by a 3D printed case, small enough to put into an incubator on top of a small stir plate.

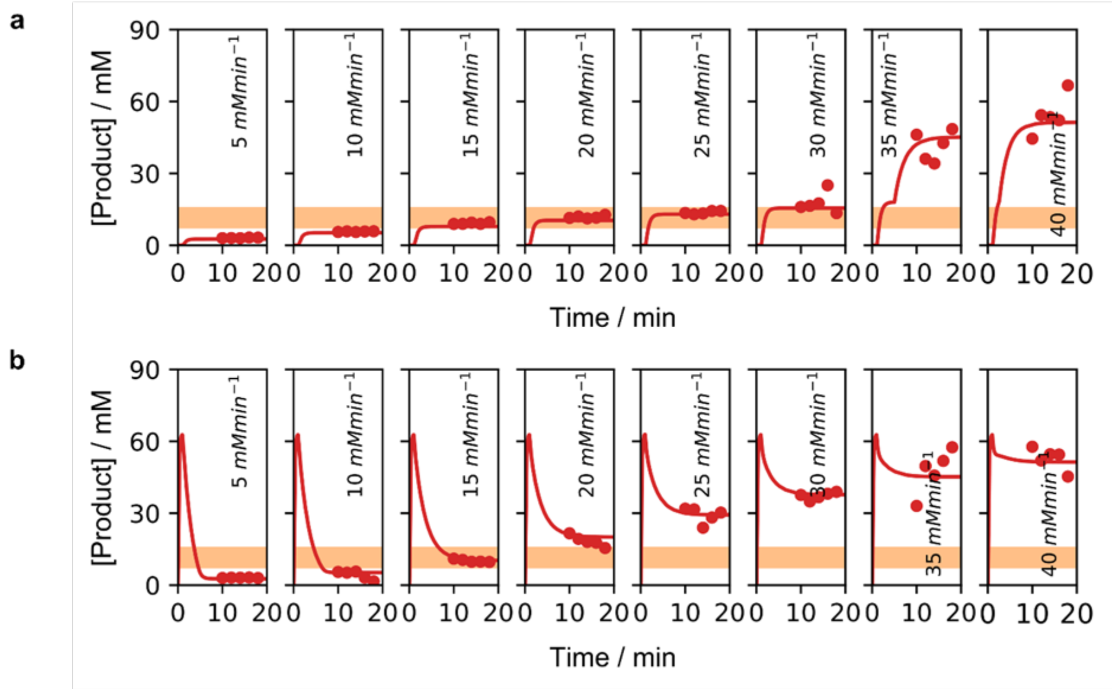

Supplementary Figure 6: The steady state concentration of the anhydride product of 1 at 24 °C and a space velocity of 0.4 min^{-1}. The markers indicate concentrations measured by HPLC and the solid line represents modelled data. **a** When fueling at least with 35 mM.min^{-1} EDC, the steady state concentration surpasses the metastable zone (marked orange) and crystal formation starts resulting in much higher steady state levels. **b** When adding an 80 mM EDC spike before starting the constant influx of EDC and precursor, crystal formation already starts at lower fuel influxes, i.e., at 20 mM.min^{-1} EDC.

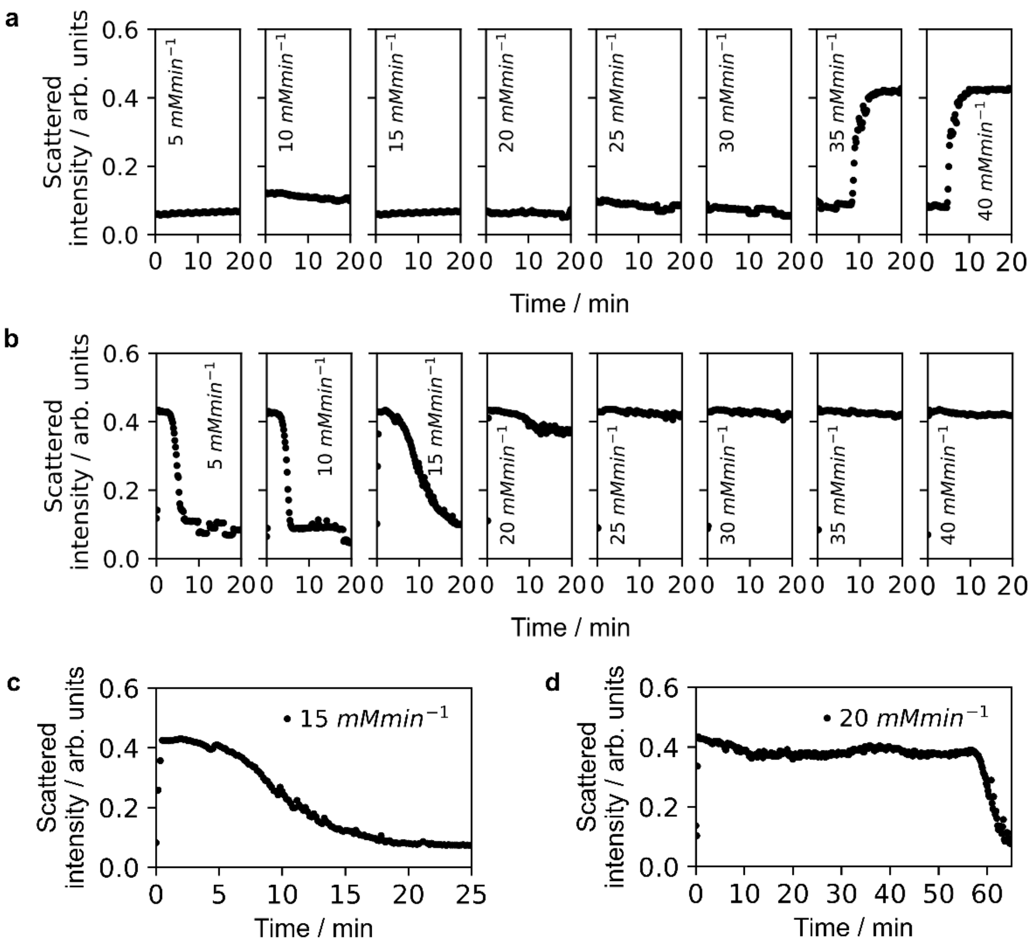

Supplementary Figure 7: The scattering of reaction cycle 1 as a measure for turbidity at 24 °C and a space velocity of 0.4 min^{-1}. a When applying at least a 35 mM.min^{-1} EDC flux, crystallization starts to occur which is shown by an increase in the scattering at 10 min. **b** The addition of an 80 mM EDC spike before starting the influx of EDC and precursor results in initial crystal formation. Low EDC fluxes are not sufficient to sustain the crystals. At least a 20 mM.min^{-1} EDC flux was required, resulting in a steady state in sustained turbidty reached at around 10 min. The continuous turbidity indicates the persistent presence of crystals. **c**, **d** The curves from b measured for longer times.

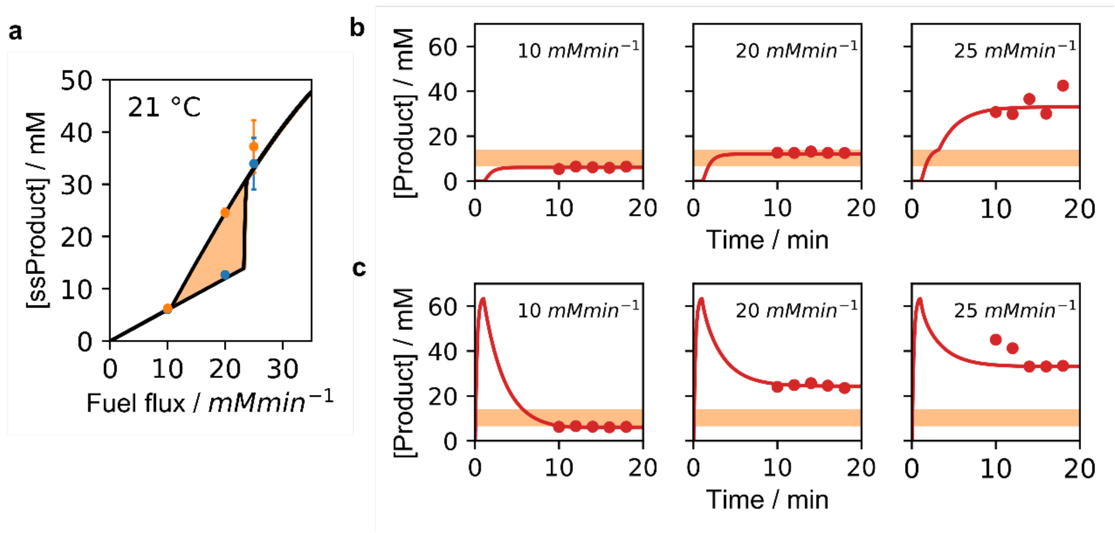

Supplementary Figure 8: The steady state concentration of the anhydride product of 1 at 21 °C and a space velocity of 0.4 min^{-1}. a The hysteresis curve was calculated with the kinetic model by incrementally increasing and decreasing the EDC fuel flux. Several points were experimentally validated by calculating the mean and standard deviation of five steady state datapoints measured by HPLC. The bistable window is marked in orange. **b** The steady state concentrations at different fuel fluxes. The markers indicate concentrations measured by HPLC and the solid line represents modelled data when no EDC and **c**, an 80 mM EDC spike was added before starting the constant influx of EDC and precursor. The orange zone represents the metastable zone.

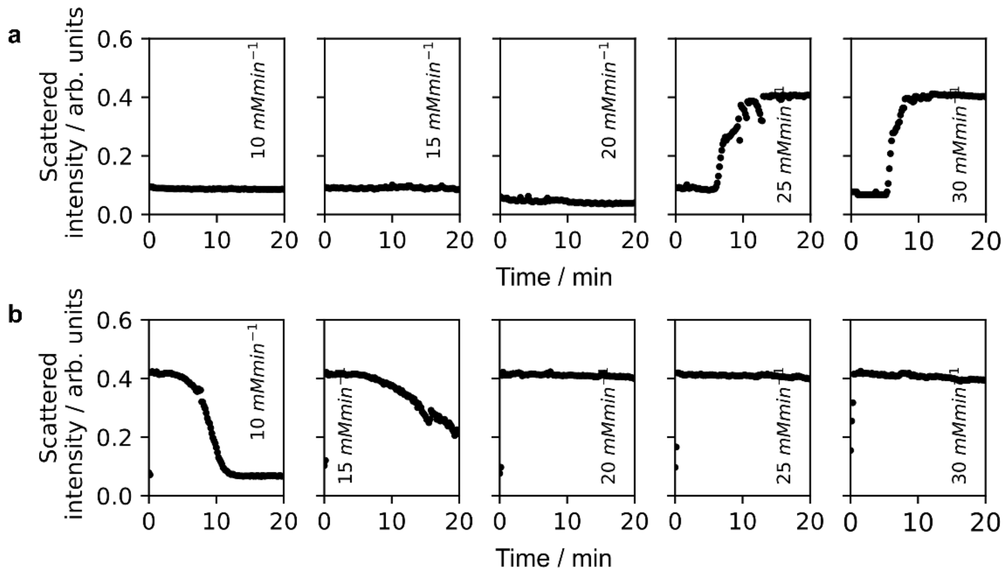

Supplementary Figure 9: The scattering of reaction cycle 1 as a measure for turbidity at 21 °C and a space velocity of 0.4 min^{-1}. a No EDC and **b**, an 80 mM EDC spike was added before starting the pumps.

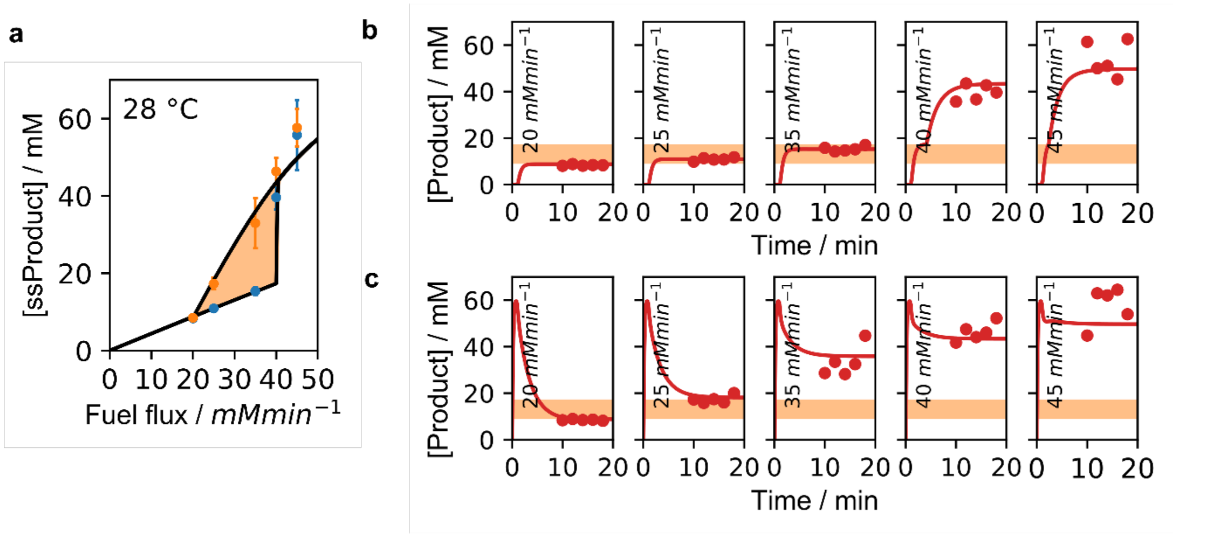

Supplementary Figure 10: The steady state concentration of the anhydride product of 1 at 28 °C and a space velocity of 0.4 min^{-1}. a The hysteresis curve was calculated with the kinetic model by incrementally increasing and decreasing the EDC fuel flux. Several points were experimentally validated by calculating the mean and standard deviation of five steady state datapoints measured by HPLC. The bistable window is marked in orange. **b** The steady state concentrations at different fuel fluxes. The markers indicate concentrations measured by HPLC and the solid line represents modelled data when no EDC and **c** an 80 mM EDC spike was added before starting the constant influx of EDC and precursor. The orange zone represents the metastable zone.

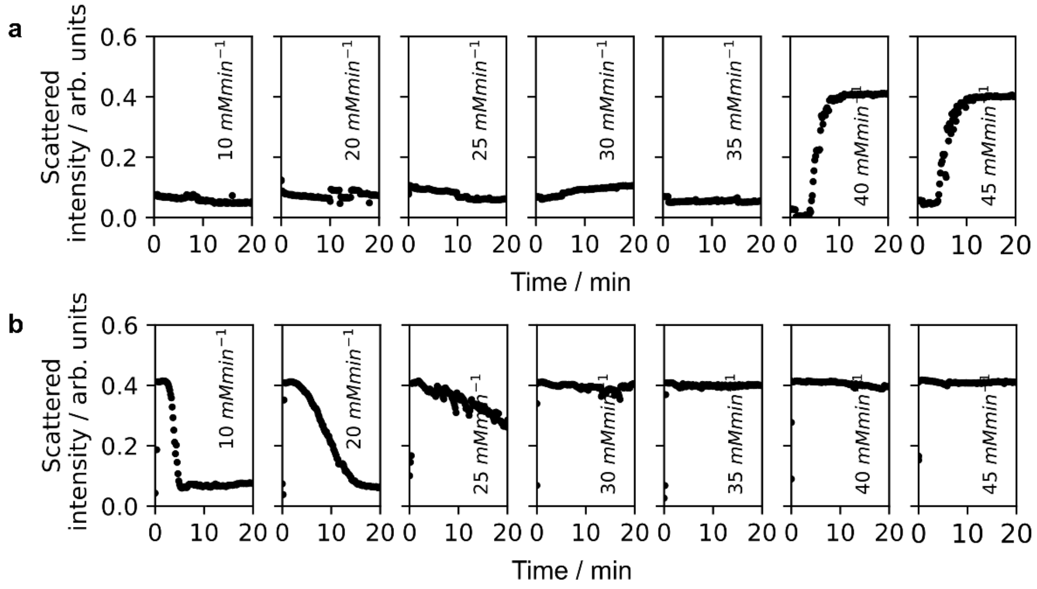

Supplementary Figure 11: The scattering of reaction cycle 1 as a measure for turbidity at 28 °C and a space velocity of 0.4 min^{-1}. a No EDC and **b** an 80 mM EDC spike was added before starting the pumps.

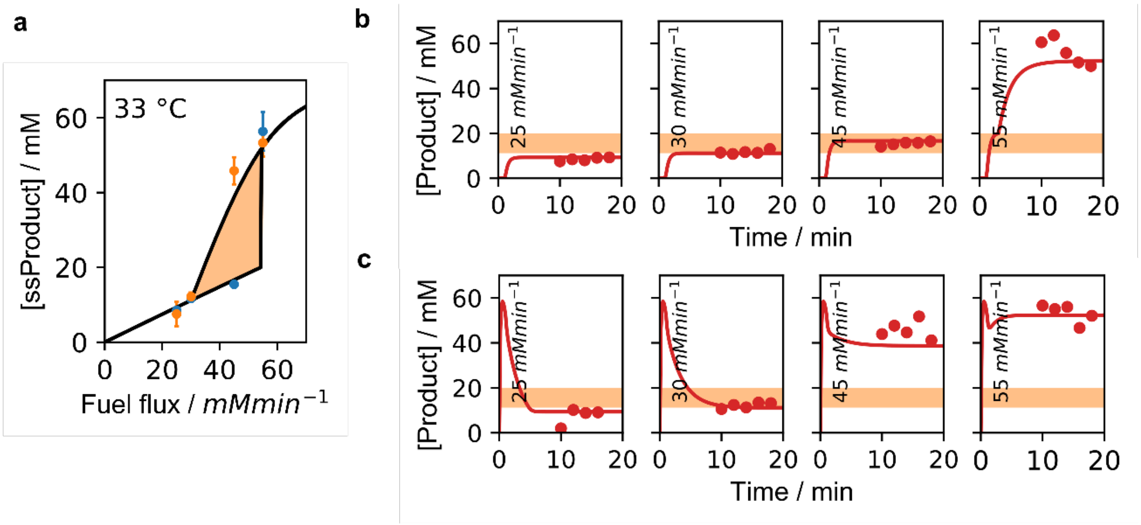

Supplementary Fig. 12: The steady state concentration of the anhydride product of 1 at 33 °C and a space velocity of 0.4 min^{-1}. a, The hysteresis curve was calculated with the kinetic model by incrementally increasing and decreasing the EDC fuel flux. Several points were experimentally validated by calculating the mean and standard deviation of five steady state datapoints measured by HPLC. The bistable window is marked in orange. **b**, The steady state concentrations at different fuel fluxes. The markers indicate concentrations measured by HPLC and the solid line represents modelled data when no EDC and **c**, an 80 mM EDC spike was added before starting the constant influx of EDC and precursor. The orange zone represents the metastable zone.

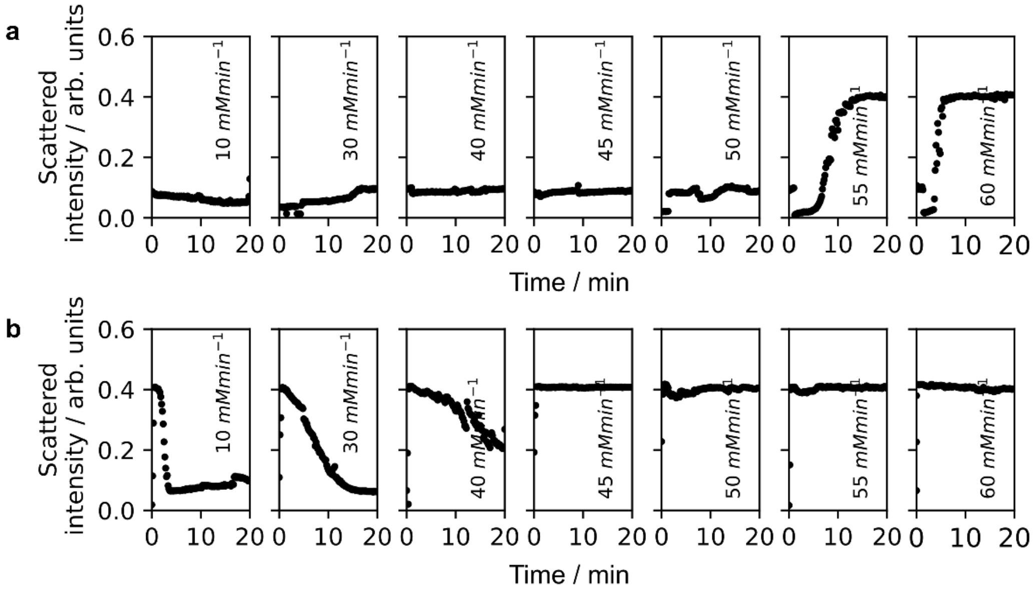

Supplementary Figure 13: The scattering of reaction cycle 1 as a measure for turbidity at 33 °C and a space velocity of 0.4 min^{-1}. a No EDC and **b**, an 80 mM EDC spike was added before starting the pumps.

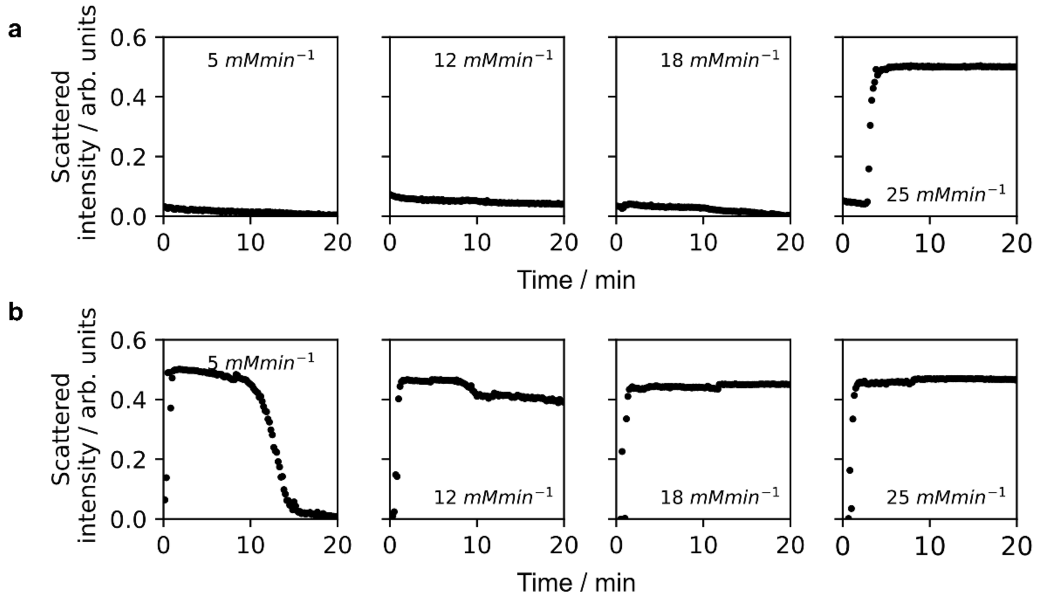

Supplementary Figure 14: The scattering of reaction cycle 1 as a measure for turbidity at 24 °C and a space velocity of 0.0 min^{-1}. A 2 ml reaction vial containing 60 mM precursor **1** was influxed with different amounts of EDC. The flowrate was set to 15 μl.min^{-1}. **a** No EDC and **b**, an 80 mM EDC spike was added before starting the pumps.

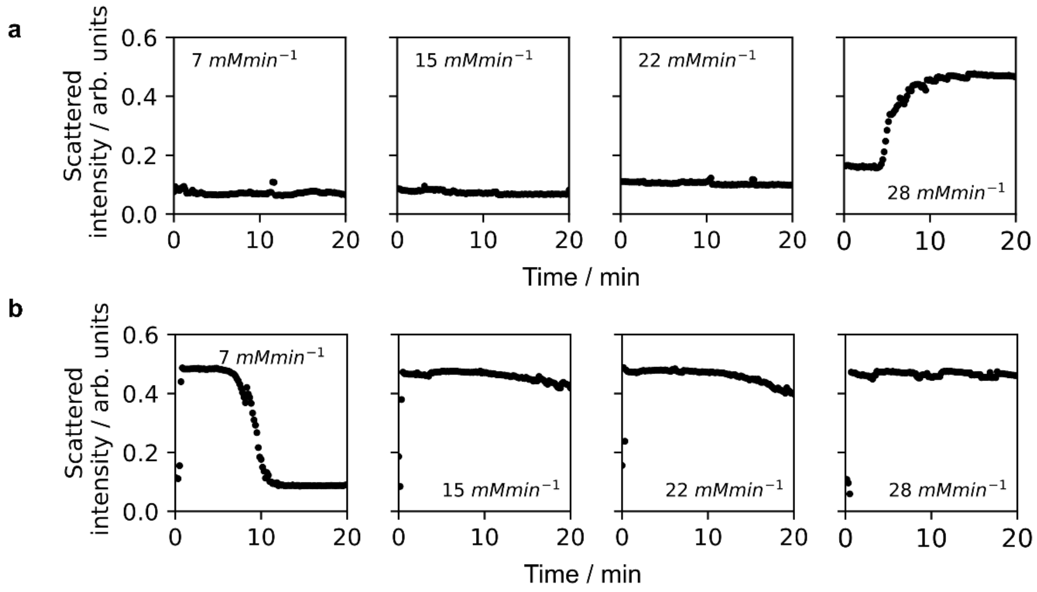

Supplementary Figure 15: The scattering of reaction cycle 1 as a measure for turbidity at 24 °C and a space velocity of 0.2 min^{-1}. The flowrate in was set to 0.15 ml.min^{-1} and the flowrate out of the reactor was set to 0.30 ml.min^{-1}. **a** No EDC and **b**, an 80 mM EDC spike was added before starting the pumps.

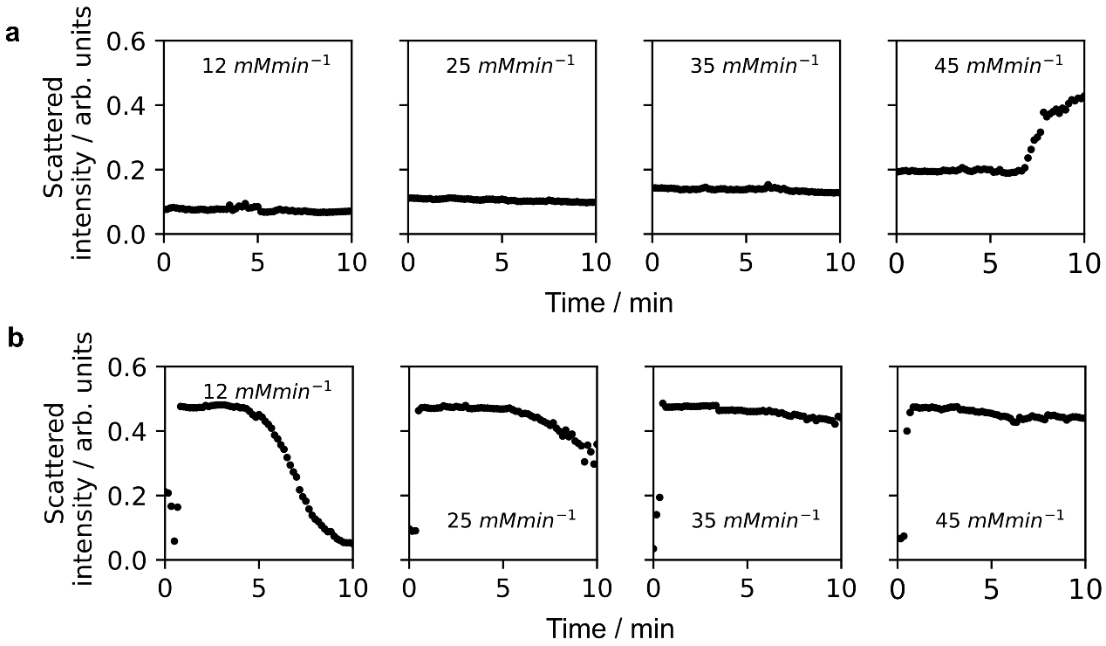

Supplementary Figure 16: The scattering of reaction cycle 1 as a measure for turbidity at 24 °C and a space velocity of 0.6 min^{-1}. The flowrate in was set to 0.45 $ml.min^{-1}$ and the flowrate out of the reactor was set to 0.90 $ml.min^{-1}$. **a** No EDC and **b**, an 80 mM EDC spike was added before starting the pumps.

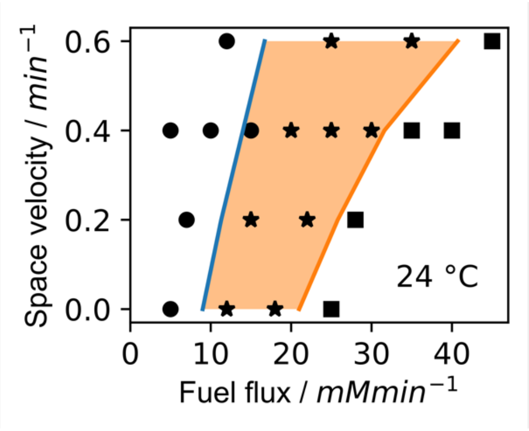

Supplementary Figure 17: The bistability window at 24 °C at various space velocities as determined by turbidity measurements. The stars mark the energy flux at which two stable steady-states were found. The blue and orange line are intersections of the low and high steady state concentrations determined by the kinetic model (Fig. 3d).

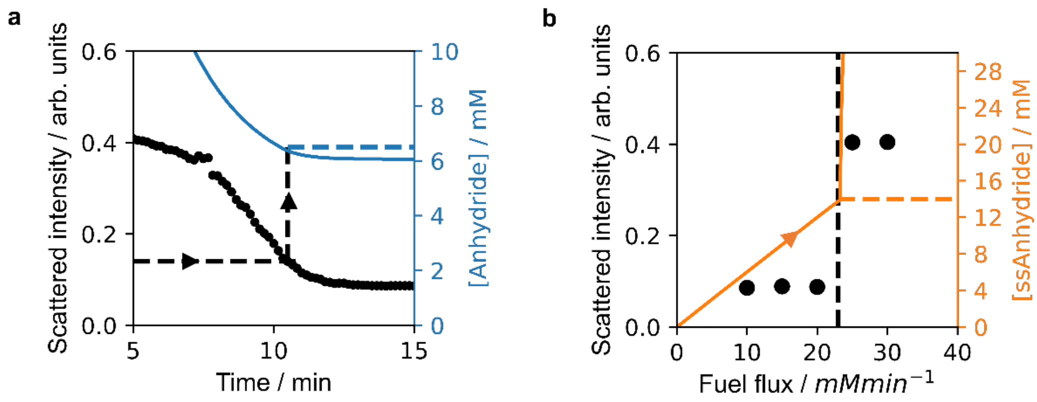

Supplementary Figure 18: Determining the temperature dependence of S_{out} and S_{sat}. The method is exemplary shown at 21 °C for all temperatures in the phase diagram (Fig. 1 c). **a** The scattering over time when a 10 mM.min^{-1} EDC flux was applied to the reaction vessel after adding an 80 mM EDC spike at 21 °C. The blue line represents the anhydride evolution over time calculated by the kinetic model and yields S_{out}. **b** The steady state scattering measured when different EDC fluxes are applied at 21 °C. The orange line represents the appearing steady state concentration in anhydride calculated by the model and yields S_{sat}.

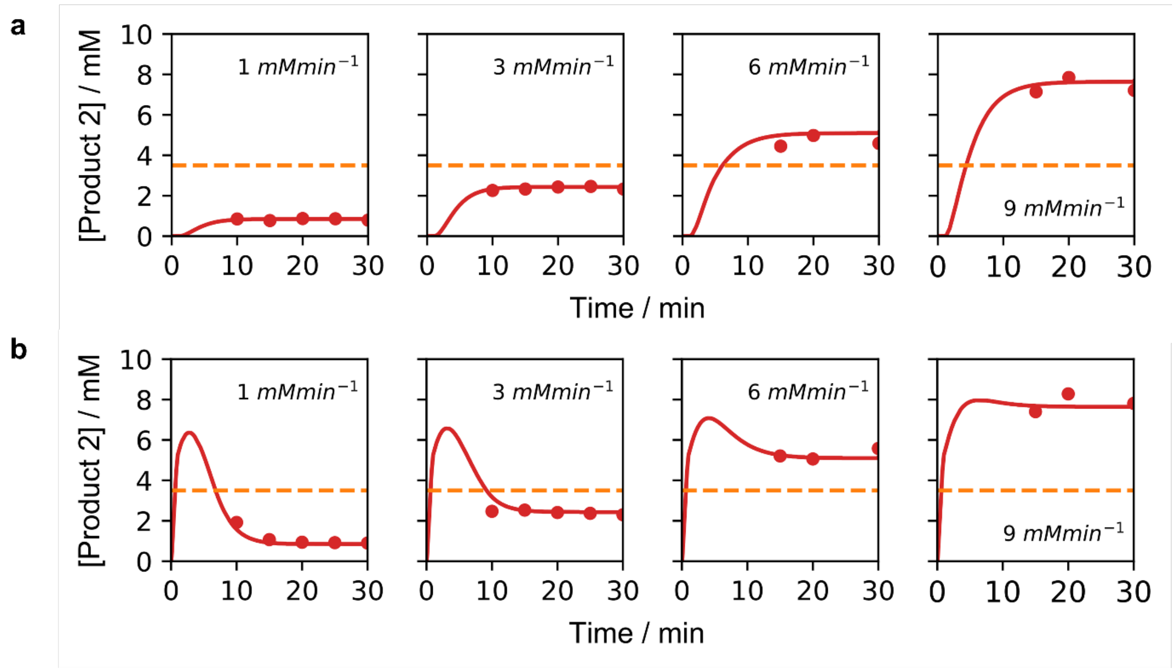

Supplementary Figure 19: The steady state concentration of the anhydride product of 2 at 25 °C, pH 6.0 and a space velocity of 0.32 min^{-1}. The markers indicate concentrations measured by HPLC and the solid line represents modelled data. Whilst the precursor influx was kept constant at 6 mM.min^{-1}, several EDC fluxes were applied when **a**, no EDC and **b**, a 50 mM EDC spike was added at the beginning.

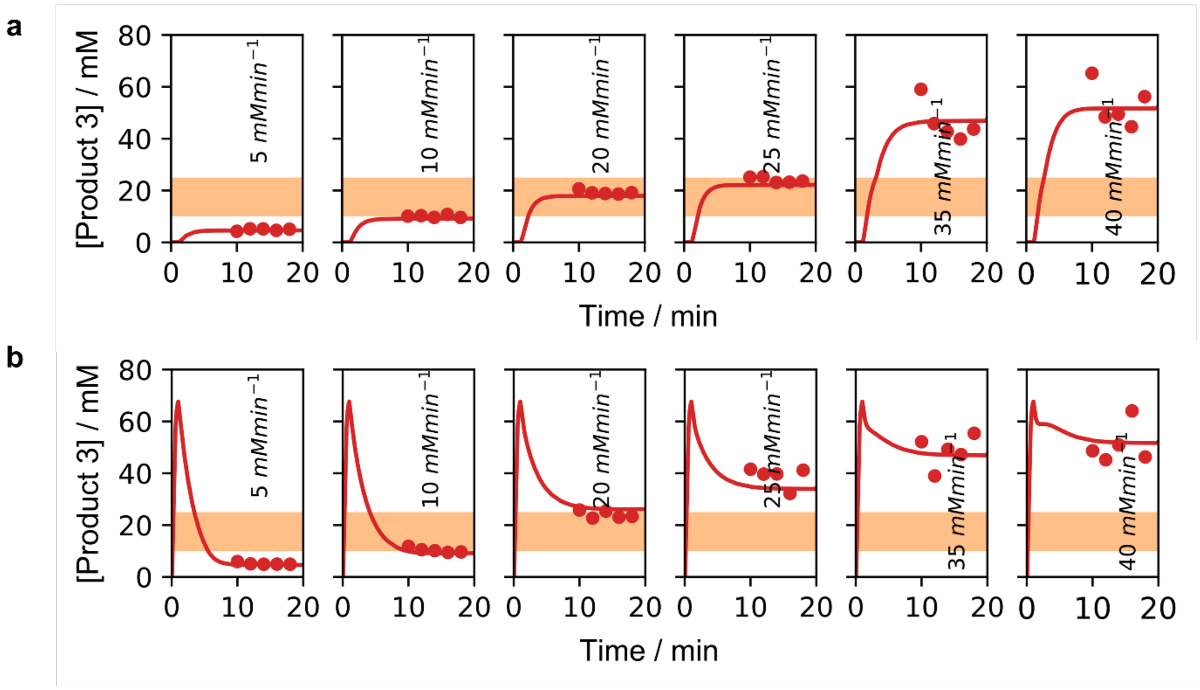

Supplementary Figure 20: The steady state concentration of the anhydride product of 3 at 24 °C, pH 3.5 and a space velocity of 0.4 min^{-1}. The markers indicate concentrations measured by HPLC and the solid line represents modelled data. The precursor was influxed continuously at a rate of 30 mM.min^{-1}. Increasing the EDC flux increases the steady state concentration and **a**, crystals start to form at an EDC flux of at least 35 mM.min^{-1}. **b** When adding an 80 mM EDC spike before starting the pumps, an EDC flux of 20 mM.min^{-1} is sufficient to form sustained crystals and results in higher anhydride steady state concentrations.

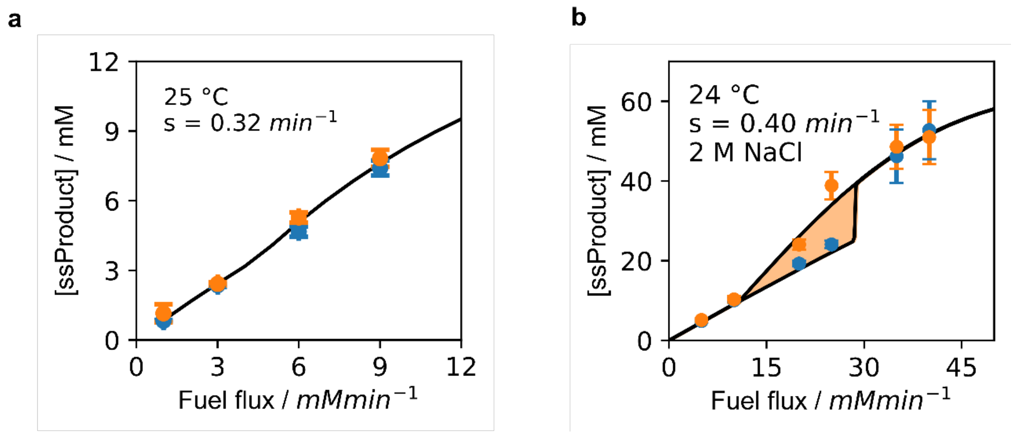

Supplementary Figure 21: Hysteresis curves of further precursors. The markers represent the measured mean steady state concentration of **a**, precursor **2** and **b**, precursor **3** and their standard deviation (*n*=5). The line is calculated by the kinetic model.

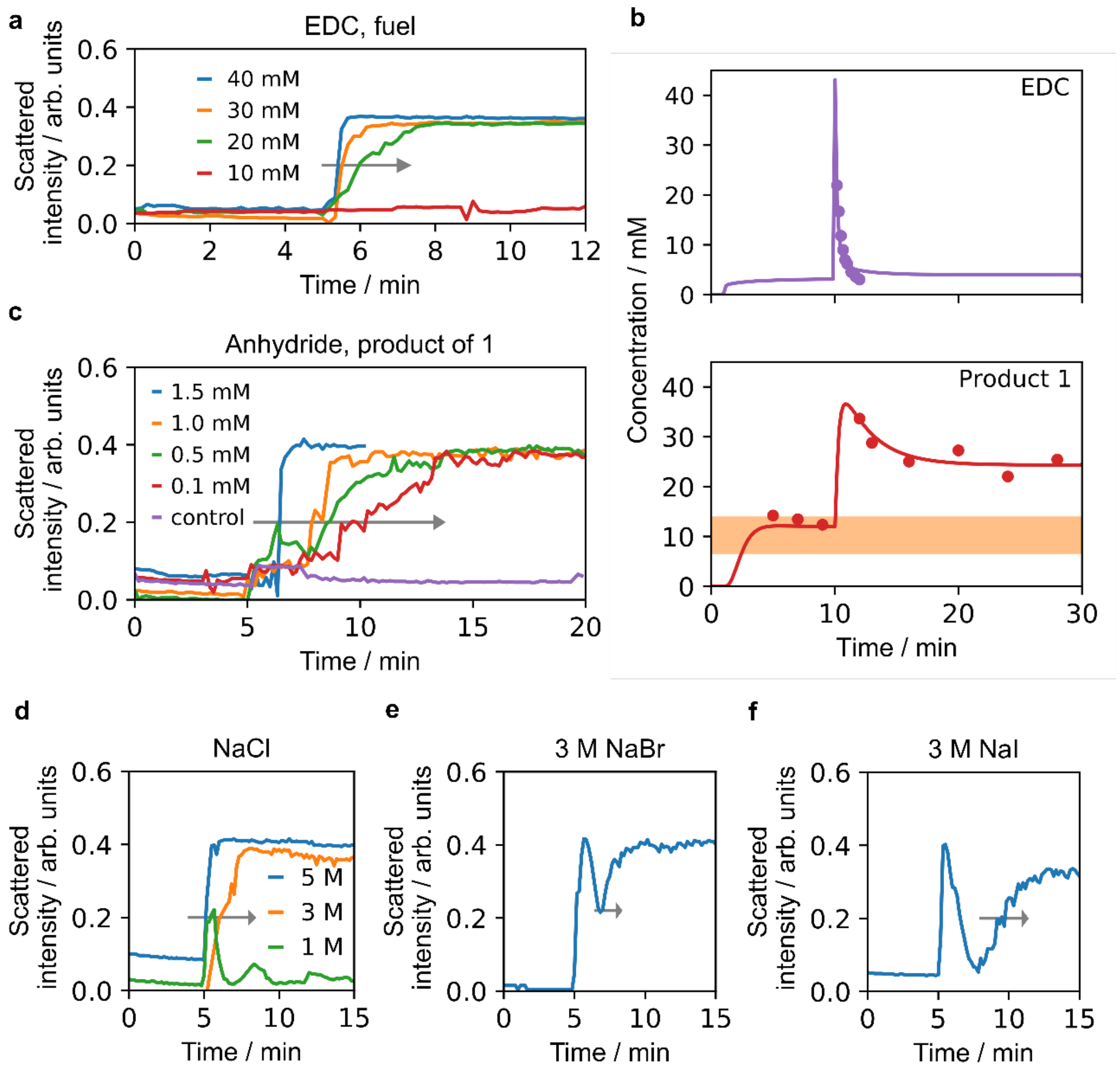

Supplementary Figure 22: Toggling the switch experiment – Activation: Various triggers were applied to switch the system on at 5 min when influxing 20 mM.min^{-1} EDC and 30 mM.min^{-1} precursor **1** at 21 °C. **a** The scattering over time taken as a measure for turbidity when adding various EDC fuel concentrations. **b** The steady state concentrations of EDC and anhydride over time as measured by HPLC and confirmed by the kinetic model. The transient addition of 40 mM EDC switches a steady state concentration inside the metastable zone to a higher steady state concentration with sustained crystal formation. **c** The scattering over time when seeding with crystalline anhydride. As negative control, polystyrene beads were added to test the effect of additional nucleation sites. **d** The effect of adding high concentration crystalline NaCl, **e**, NaBr and **f**, NaI.

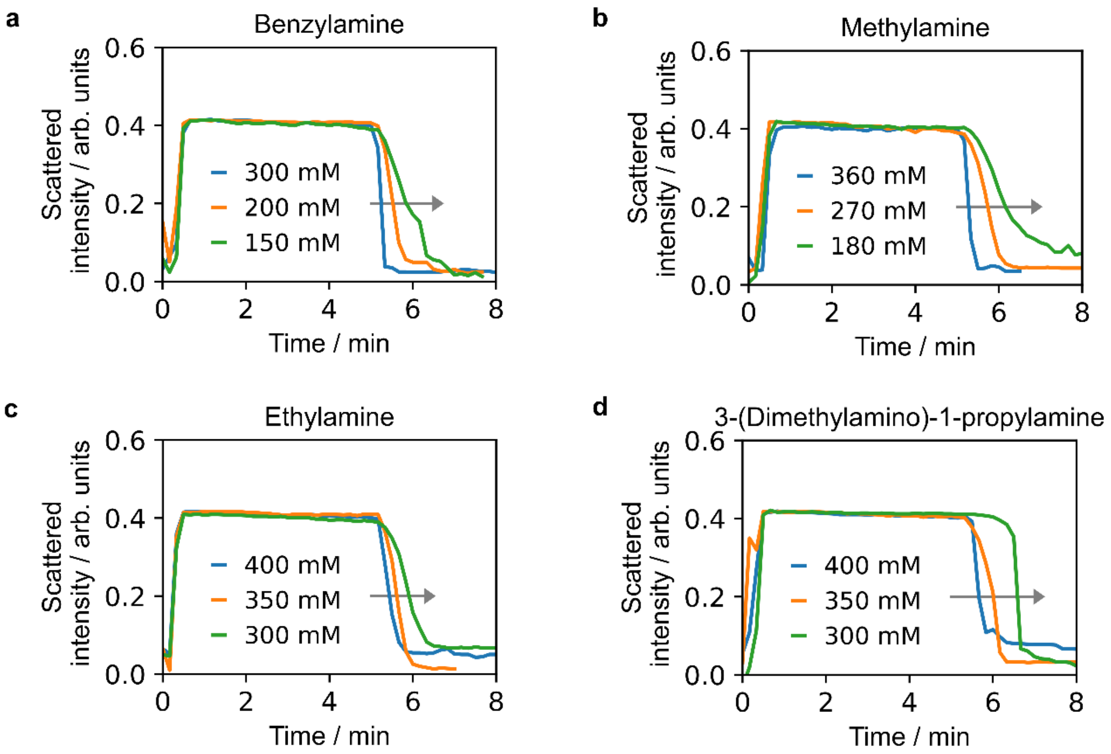

Supplementary Figure 23: Toggling the switch experiment - Deactivation. The system was influxed with 20 mM.min^{-1} EDC and 30 mM.min^{-1} precursor **1**. Spiking with 40 mM EDC before starting the pumps led to a sustained on state. At 5 min **a**, benzylamine, **b**, methylamine, **c**, ethylamine and **d,** a propylamine derivative was added as an off trigger. The decrease in scattering was measured over time to estimate the effectivness of deactivation.

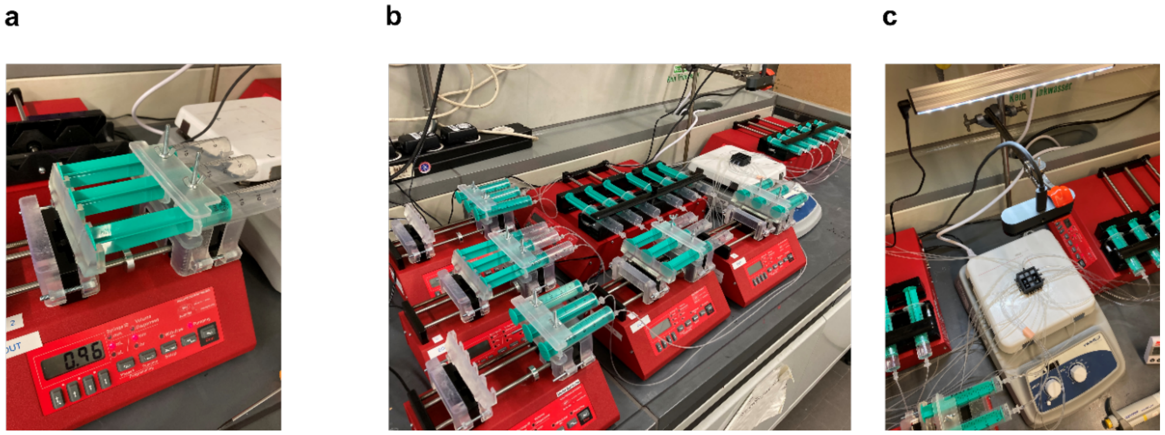

Supplementary Figure 24: The experimental setup of writing pixel-based patterns. a The 3D printed extension allows to control three syringes with one syringe pump. **b** To influx and outflux one reactor, three syringes are needed giving a total number of 27 syringes for a 9-digit pixel display. **c**, The 3x3 array was placed on a stir plate and timelapse photographs were taken by a webcam.

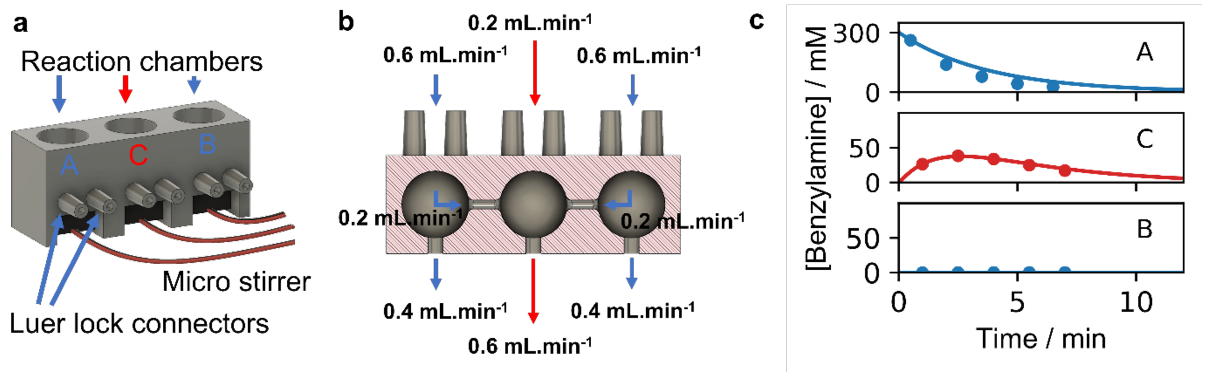

Supplementary Figure 25: A 3D printed setup to combine three switches to an OR-port. **a** The reactors A and B correspond to the input reactor and the middle reactor C serves as a readout. Each reactor is individually fueled with precursor **1** and EDC under a constant outflux. **b** A connection between the reactors allows an influx from the outer reactors into the middle reactor. **c** The influx of reactor A to C is tested by adding 300 mM of benzylamine to reactor A and monitoring the concentration over time by HPLC (marker). The line is predicted by the Matlab model.

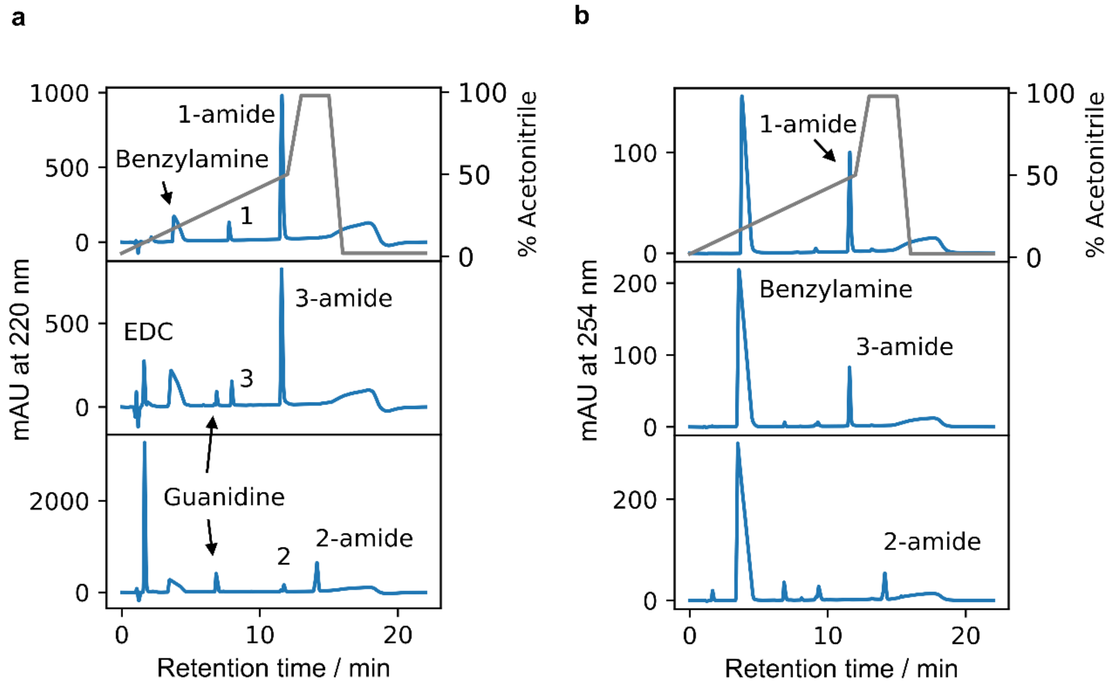

Supplementary Figure 26: The HPLC chromatograms of the quenched reaction cycle. Retention times and pump gradient of cycle **1**, **3** and **2** as recorded at **a**, 220 nm and **b**, 254 nm.

Supplementary Tables

	precursor **1**				precursor **2**[3]	precursor **3**
	21 °C	24 °C	28 °C	33 °C	25 °C	24 °C
k_1 ($M^{-1}s^{-1}$)	0.500				0.200	0.450
k_2 (s^{-1})	$0.600*k_1$	$1.14*k_1$	$1.20*k_1$	$1.60*k_1$	$1.00*k_1$	$0.150*k_1$
k_3 (s^{-1})	$0.0500*k_1$				$0.250*k_1$	$0.0100*k_1$
k_4 (s^{-1})	(0.00130*Temperature) – 0.0113				0.00350	0.00740

Supplementary Table 1: The rate constants used in the kinetic model. The deactivation rate constant k_4 of precursor **1** and **2** was extracted from batch experiments (SI 2). The rate constant for the activation (k_1) at 25 °C of precursor **1** was experimentally determined in previous work by monitoring the EDC decay profile with HPLC[4]. Its temperature dependence was estimated by fitting the previously mentioned batch experiments. The rate constants for k_2 and k_3 are not experimentally accessible. Their values were determined by fitting the experimental data and listed as a multiple of k_1.

X-ray Crystallographic Details

Single crystals were grown by dissolving synthesized and purified[4] anhydride of precursor **1** in minimum amount of acetone. Thereafter, the solution was filtered through a PTFE syringe filter. Crystals were obtained by diffusion of pentane into the acetone solution.

Data were collected on a single crystal x-ray diffractometer equipped with a CMOS detector (Bruker Photon-100), a TXS rotating anode with MoKα radiation (λ = 0.71073 Å) and a Helios optic using the APEX3 software package[5]. The measurements were performed on single crystals coated with perfluorinated ether. The crystals were fixed on top of a kapton micro sampler and frozen under a stream of cold nitrogen. A matrix scan was used to determine the initial lattice parameters. Reflections were corrected for Lorentz and polarisation effects, scan speed, and background using SAINT[6]. Absorption correction, including odd and even ordered spherical harmonics was performed using SADABS[6]. Space group assignment was based upon systematic absences, E statistics, and successful refinement of the structure. The structures were solved using SHELXT with the aid of successive difference Fourier maps, and were refined against all data using SHELXL in conjunction with SHELXLE[7,8,9]. Hydrogen atoms

(except on heteroatoms) were calculated in ideal positions as follows: Methyl hydrogen atoms were refined as part of rigid rotating groups, with a C–H distance of 0.98 Å and Uiso(H) = 1.5·Ueq(C). Non-methyl H atoms were placed in calculated positions and refined using a riding model with methylene, aromatic, and other C–H distances of 0.99 Å, 0.95 Å, and 1.00 Å, respectively, and Uiso(H) = 1.2·Ueq(C). Non-hydrogen atoms were refined with anisotropic displacement parameters. Full-matrix least-squares refinements were carried out by minimizing Σw(Fo2 Fc2)2 with the SHELXL weighting scheme.3 Neutral atom scattering factors for all atoms and anomalous dispersion corrections for the non-hydrogen atoms were taken from International Tables for Crystallography[10]. Images of the crystal structure were generated with PLATON[11]. Deposition Number 2127541 contains the supplementary crystallographic data for this paper. These data are provided free of charge by the joint Cambridge Crystallographic Data Centre and Fachinformationszentrum Karlsruhe Access Structures service www.ccdc.cam.ac.uk/structures.

Anhydride product 1 (CCDC 2127541)

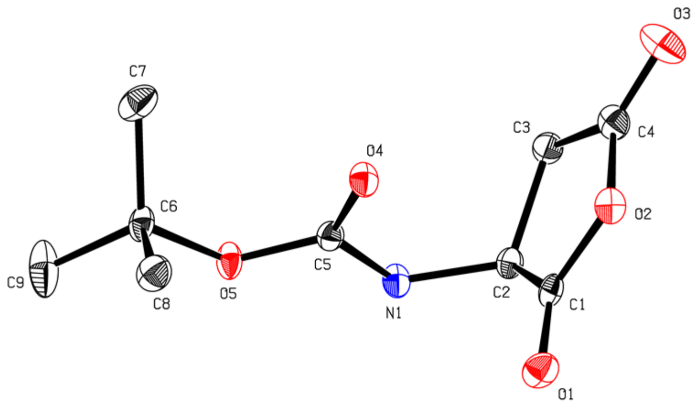

Diffractometer operator C. Jandl
scanspeed 1-5 s per frame
dx 50 mm
2311 frames measured in 8 data sets
phi-scans with delta_phi = 0.5
omega-scans with delta_omega = 0.5
shutterless mode

Crystal data

$C_9H_{13}NO_5$	
M_r = 215.20	D_x = 1.384 Mg m^{-3}
Orthorhombic, $P2_12_12_1$	Melting point: ? K
Hall symbol: P 2ac 2ab	Mo *K*α radiation, λ = 0.71073 Å

a = 5.1839 (6) Å	Cell parameters from 9496 reflections
b = 17.5000 (19) Å	θ = 2.5–26.4°
c = 22.773 (3) Å	μ = 0.11 mm^{-1}
V = 2065.9 (4) $Å^3$	T = 100 K
Z = 8	Fragment, colourless
F(000) = 912	0.33 × 0.16 × 0.08 mm

Data collection

Bruker Photon CMOS diffractometer	4252 independent reflections
Radiation source: TXS rotating anode	4104 reflections with $I > 2\sigma(I)$
Helios optic monochromator	R_{int} = 0.032
Detector resolution: 16 pixels mm^{-1}	θ_{max} = 26.4°, θ_{min} = 2.1°
phi– and ω–rotation scans	h = −6 6
Absorption correction: multi-scan *SADABS* 2016/2, Bruker	k = −21 21
T_{min} = 0.705, T_{max} = 0.745	l = −28 28
55658 measured reflections	

Refinement

Refinement on F^2	Hydrogen site location: mixed
Least-squares matrix: full	H atoms treated by a mixture of independent and constrained refinement
$R[F^2 > 2\sigma(F^2)]$ = 0.026	W = $1/[\Sigma^2(FO^2) + (0.0342P)^2 + 0.4152P]$ WHERE $P = (FO^2 + 2FC^2)/3$
$wR(F^2)$ = 0.065	$(\Delta/\sigma)_{max}$ < 0.001
S = 1.11	$\Delta\rho_{max}$ = 0.18 e $Å^{-3}$
4252 reflections	$\Delta\rho_{min}$ = −0.21 e $Å^{-3}$
285 parameters	Extinction correction: none
0 restraints	Extinction coefficient: -
0 constraints	Absolute structure: Flack, Parsons[8]
Primary atom site location: iterative	Absolute structure parameter: −0.14 (15)
Secondary atom site location: difference Fourier map	

Supplementary References

1. Kulkarni, S.A., Kadam, S.S., Meekes, H., Stankiewicz, A.I. & ter Horst, J.H. Crystal Nucleation Kinetics from Induction Times and Metastable Zone Widths. *Crystal Growth & Design* **13**, 2435-2440 (2013).
2. Tena-Solsona, M. et al. Non-equilibrium dissipative supramolecular materials with a tunable lifetime. *Nat Commun* **8**, 15895 (2017).
3. Wanzke, C., Tena-Solsona, M., Rieß, B., Tebcharani, L. & Boekhoven, J. Active droplets in a hydrogel release drugs with a constant and tunable rate. *Materials Horizons* **7**, 1397-1403 (2020).
4. Schnitter, F. & Boekhoven, J. A Method to Quench Carbodiimide-Fueled Self-Assembly. *ChemSystemsChem* **3** (2020).
5. *APEX suite of crystallographic software*, APEX 3, Version 2019-1.0, Bruker AXS Inc., Madison, Wisconsin, USA, 2019.
6. *SAINT*, Version 8.40A and *SADABS*, Version 2016/2, Bruker AXS Inc., Madison, Wisconsin, USA, 2016/2019.
7. G. M. Sheldrick, *Acta Crystallogr. Sect. A* **2015**, *71*, 3-8.
8. G. M. Sheldrick, *Acta Crystallogr. Sect. C* **2015**, *71*, 3-8.
9. C. B. Hübschle, G. M. Sheldrick, B. Ditrrich, *J. Appl. Cryst.* **2011**, *44*, 1281-1284
10. *International Tables for Crystallography*, *Vol. C* (Ed.: A. J. Wilson), Kluwer Academic Publishers, Dordrecht, The Netherlands, **1992**, Tables 6.1.1.4 (pp. 500-502), 4.2.6.8 (pp. 219-222), and 4.2.4.2 (pp. 193-199).
11. A. L. Spek, *Acta Crystallogr. Sect. D* **2009**, *65*, 148-155.
