# Supplementary material for: Memory, switches, and an OR-port through bistability in chemically fueled crystals
Source: Nat Commun. 2022 May 20;13:2816. doi: 10.1038/s41467-022-30424-2 (PMC9122941; doi:10.1038/s41467-022-30424-2)
Supplement: Supplementary file 3 — Description of Additional Supplementary Files [file 41467_2022_30424_MOESM3_ESM.pdf]

Description of Additional Supplementary Files

Supplementary Movie 1: Toggling between off and on.

Supplementary Movie 2: Pixel display.

Supplementary Movie 3: Logic OR-gate.
